# Breed and Feeding System Impact the Bioactive Anti-Inflammatory Properties of Bovine Milk

**DOI:** 10.3390/ijms231911088

**Published:** 2022-09-21

**Authors:** Angela Salzano, Maria Chiara Di Meo, Nunzia D’Onofrio, Giovanna Bifulco, Alessio Cotticelli, Francesca Licitra, Antonio Iraci Fuintino, Giuseppe Cascone, Maria Luisa Balestrieri, Ettore Varricchio, Giuseppe Campanile

**Affiliations:** 1 Department of Veterinary Medicine and Animal Productions, University of Naples Federico II, 80137 Naples, Italy; 2 Department of Science and Technology, University of Sannio, 82100 Benevento, Italy; 3 Department of Precision Medicine, University of Campania Luigi Vanvitelli, 80138 Naples, Italy; 4 Istituto Zooprofilattico Sperimentale della Sicilia “A. Mirri”, 90129 Palermo, Italy; 5 Provincial Public Health Authority of Ragusa, 97100 Ragusa, Italy

**Keywords:** dairy cattle, Modicana, milk, antioxidant, betaine

## Abstract

In the present study, we aimed at assessing the influence of breed and feeding system on the bovine milk profile of betaines and carnitines and milk capacity in counteracting the inflammatory endothelial cell (EC) damage induced by interleukin (IL)-6. In the first experimental design, two breeds were chosen (*Holstein* vs. *Modicana*) to investigate the biomolecule content and antioxidant capacity in milk and dairy products. In the second experimental design, two feeding systems (pasture vs. total mixed ratio) were tested only in *Holstein* to evaluate the possible effect on the functional profile of milk and dairy products. Finally, the bulk milk from the two experimental designs was used to evaluate the efficacy of preventing IL-6-induced endothelial inflammatory damage. Results showed that *Modicana* milk and whey had higher biomolecule content and antioxidant activity compared to *Holstein* milk (*p* < 0.01). Milk from *Holstein* fed TMR showed higher concentration of γ-butyrobetaine, δ-valerobetaine (*p* < 0.01), and l-carnitine (*p* < 0.05). Similarly, whey from *Holstein* fed TMR also showed higher content of δ-valerobetaine, glycine betaine, l-carnitine, and acetyl-l-carnitine (*p* < 0.01) compared to the *Holstein* fed pasture. Conversely, the antioxidant activity of milk and dairy products was not affected by the feeding system. In ECs, all milk samples reduced the IL-6-induced cytokine release, as well as the accumulation of reactive oxygen species (ROS) and the induction of cell death, with the most robust effect elicited by *Modicana* milk (*p* < 0.01). Overall, *Modicana* milk showed a higher content of biomolecules and antioxidant activity compared to *Holstein*, suggesting that the breed, more than the feeding system, can positively affect the health-promoting profile of dairy cattle milk.

## 1. Introduction

Local breeds are more adaptable to different habitats compared to cosmopolitan breeds in terms of disease resistance, heat tolerance, and low nutrition needs [1]. Moreover, their productions have higher sensorial, nutritive, and technological properties arising from the feeding and keeping system [1]. For these reasons, they represent a significant genetic and economic resource that the European Union policy wants to preserve. In Italy, most of the reared breeds are the cosmopolite specialized ones, but several local cattle breeds are still bred in marginal areas, mostly in extensive and semi-intensive systems [2]. Within Italian autochthonous breeds, the *Modicana* is mainly reared in Sicily, in the Hyblaean plateau, for milk and cheese production. In particular, with *Modicana*’s milk, it is possible to produce a cheese with a protected designation of origin (PDO) label, named “Ragusano”, aging from 4 to 12 months. *Modicana* cows are usually reared in extensive systems, using pasture during the grazing season and with limited or no supplementation of concentrate. Semi-intensive farming systems are also possible when animals cannot go to pasture and need higher concentrate supplementation [3,4]. Feeding has an important role in determining most milk quality traits such as fat content and fatty acid composition [5]. Indeed, pasture-based diets can confer characteristic aroma, color, and biologically active and health-promoting biomolecules in milk to both milk and dairy products [6,7,8].

Among healthy molecules, betaine and carnitine precursors are broadly present in ruminant meat and milk [9] and are both essential in human health [10] due to their antioxidant, anti-inflammatory, and anticancer properties [11,12,13,14,15]. However, among ruminants, bovine milk has a lower concentration of carnitine and betaine compared to buffalo milk [16] but, in the latter study, only *Holstein* milk was used to assess the concentration of biomolecules. At present, the concentrations of carnitine and betaine in all the other breeds are still unknown. With the increased demand for “functional” and “natural” foods, several studies have highlighted the feasibility of modifying milk composition at the farm stage through breeding techniques, without the need for further mechanical modification [7,17,18]. In buffaloes, it has been previously demonstrated that higher space availability (>15 m^2^/head) [19] or the inclusion of 30% of green feed in the diet [20] led to a greater concentration of biomolecules in milk and in dairy products. The presence of functional molecules in milk and dairy products of local breeds such as *Modicana*, with beneficial effects for human health, could promote their niche products in terms of quality and protect these breeds from extinction, since their low production remains the limiting factor that endangers their existence.

The content of healthy molecules in milk and dairy products is of fundamental importance, since several consumers have adopted a dairy-free diet due to suggested associations between milk and dairy products with obesity and coronary diseases [21]. However, in this regard, the data are conflicting, as a growing number of studies have demonstrated [22,23] that milk and dairy products have beneficial effects and an inverse association with cardiovascular disease (CVD) [24].

CVD is the main complication in type 2 diabetes mellitus (T2D), but clinical CVD can also precede the development of diabetes, supporting the hypothesis that T2D and CVD arise from common antecedents. Insulin resistance has been considered a plausible candidate for this common antecedent, but specific mechanisms whereby insulin resistance leads to T2D and CVD remain unresolved. Inflammatory cytokines such as interleukin (IL)-6 have been found to be involved in the pathogenesis of both insulin resistance and atherosclerosis [25]. Increased levels of circulating IL-6 are associated with predisposition to T2D, insulin resistance, and T2D-associated vascular complications [26]. The cellular mechanism whereby inflammation may promote these dysfunctional processes is the induction of endothelial dysfunction, indicating the vascular endothelium as a key player in the pathogenesis of CVD and T2D.

Given the health-promoting benefits of betaine and carnitine, which occur naturally in ruminant milk, it is important to establish if the concentrations of these significant functional nutrients can vary among bovine breeds or among different feeding systems. Within this framework, the present study aimed at investigating whether breed, *Modicana* (Mod) vs. *Holstein* (Hol) or, limited to Hol, the feeding system, pasture (P) vs. total mixed ratio (TMR), could influence the metabolomic profile, the antioxidant activity, and the anti-inflammatory propriety of milk.

## 2. Results

### 2.1. Influence of Breed on the Biomolecule Content and Antioxidant Activity of Milk and Dairy Products

#### 2.1.1. Average Milk Production

The milk yield was statistically different between the two breeds (3150 ± 104 kg in Mod P vs. 5300 ± 490 kg in Hol P; *p* < 0.01).

#### 2.1.2. Functional Profile and Antioxidant Activity of Bovine Milk and Dairy Products

Mod P milk showed higher concentration of all dosed bioactive compounds compared to Hol P milk (*p* < 0.01, except for acetyl- l-carnitine with *p* < 0.05; Table 1). The same pattern has been recorded for whey, in which most of the compounds were higher in Mod P compared to Hol P (*p* < 0.01). As for ricotta cheese, Mod P had higher values of γ-butyrobetaine, acetyl-l-carnitine, and propionyl-l-carnitine compared to Hol P (*p* < 0.05), while mozzarella cheese showed only a higher content of γ-butyrobetaine in Mod P compared to Hol P (*p* < 0.01).

Moreover, the total antioxidant activity (TAC) and the ferric reducing antioxidant power assay (FRAP) were always higher in Mod P milk and dairy products compared to Hol P (*p* < 0.01; Table 2).

### 2.2. Influence of Feeding System, Limited to Holstein, on the Biomolecule Content and Antioxidant Activity of Milk and Dairy Products

#### 2.2.1. Average Milk Production

No differences were found within Hol for the two different feeding systems regarding the monthly average milk production (5740 ± 350 kg vs. 5300 ± 490 kg, respectively, for Hol TMR vs. Hol P).

#### 2.2.2. Biomolecule Content and Antioxidant Activity of Milk and Dairy Products

Hol TMR showed higher concentrations of milk γ-butyrobetaine, δ-valerobetaine (*p* < 0.01), and l-carnitine (*p* < 0.05), compared to Hol P. Among dairy products, Hol TMR showed higher concentrations of whey δ-valerobetaine, glycine betaine, l-carnitine, and acetyl-l-carnitine (*p* < 0.01) compared to Hol P. In contrast, the feeding system did not affect any parameter in mozzarella and ricotta cheese (Table 3).

Conversely, TAC and FRAP were not affected by the feeding system both in milk and dairy products (Table 4).

### 2.3. Milk Extract Effects on Cell Viability

**Figure 1:**
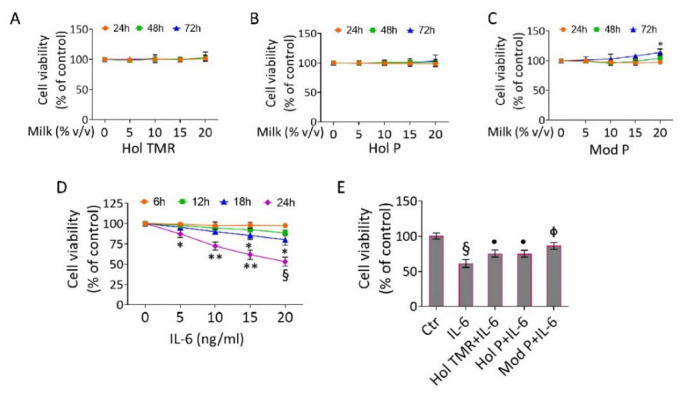
Milk extract effects on EC viability. EC viability was assayed after exposure to different volumes (0–20% *v*/*v*) of (**A**) Hol TMR, (**B**) Hol P, and (**C**) Mod P milk up to 72 h or to (**D**) different concentrations (0–20 ng/mL) of IL-6 up to 24 h or (**E**) pretreated for 16 h with milk (20% *v*/*v*) before starting stimulation for 24 h with IL-6 (20 ng/mL). Different colors and symbols mean different times of milk treatment. Cell viability was assessed by Cell Counting Kit-8 assay (Donjindo Molecular Technologies) and reported as % of control of n = 5 independent experiments. Control cells (0 or Ctr) were treated with the corresponding highest volume of HBSS-10 mM Hepes: * *p* < 0.05 vs. 0 mM or Ctr; ** *p* < 0.01 vs. 0 mM or Ctr; § *p* < 0.001 vs. 0 mM or Ctr; • *p* < 0.05 vs. IL-6; Φ *p* < 0.01 vs. IL-6.

The functional effect of the milk produced by the animals of the two experimental designs was tested in vitro on ECs. Results showed the absence of cytotoxicity up to 72 h of treatment (up to 20% *v*/*v*) (Figure 1A–C). In addition, the CCK-8 assay indicated an increased cell viability rate, with the highest effect when ECs were incubated for 72 h with Mod P milk (20% *v*/*v*) (*p* < 0.05 vs. Ctr) (Figure 1C). As shown in Figure 1D, exposure to IL-6 (20 ng/mL) resulted in 45% inhibition of cell viability (*p* < 0.01 vs. Ctr). The time-course experiments of pre-incubation with milk before inflammatory induction with IL-6 indicated that 16 h was the most effective time in preventing the reduction in cell viability induced by IL-6 (Figure S1). Hol TMR, Hol P, and Mod P milk (20% *v*/*v*) were able to counteract the decrease in cell viability induced by IL-6 (Figure 1E). In detail, pre-treatment for 16 h with 20% *v/v* of Hol TMR and Hol P determined a recovery (+14%) of cell viability rate (*p* < 0.05 vs. IL-6). Mod P milk (20% *v*/*v*) displayed the highest protective capacity (+25% vs. IL-6, *p* < 0.01) (Figure 1E). The effects of milk and IL-6 treatments on EC viability were also reported as optical density (O.D.) values. Based on these results, further cell-based experiments were performed with 20% *v/v* of Hol TMR, Hol P, and Mod P milk and 16 h of pre-incubation time before IL-6 stimulation.

### 2.4. Milk Extract Effects on Inflammation

**Figure 2:**
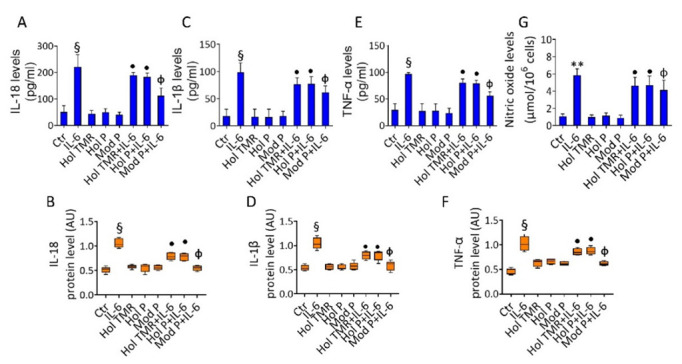
Milk extract effects on inflammation. Cytokine content and protein expression of (**A**,**B**) IL-18, (**C**,**D**) IL-1β, and (**E**,**F**) TNF-α, detected by ELISA assay and Western blotting analysis. (**G**) Nitric oxide content evaluated in ECs treated with Hol TMR, Hol P, or Mod P milk (20% *v*/*v*) for 72 h, IL-6 (20 ng/mL) for 24 h, or pretreated for 16 h with milk before stimulation for 24 h with IL-6. Control cells (Ctr) were treated with the corresponding highest volume of HBSS-10 mM Hepes. For immunoblotting, boxplots represent the densitometric mean values, expressed as arbitrary units (AU) of n = 5 independent experiments: ** *p* < 0.01 vs. Ctr; § *p* < 0.001 vs. Ctr; • *p* < 0.05 vs. IL-6; Φ *p* < 0.01 vs. IL-6.

The capability of milk in counteracting the IL-6-related inflammatory pathway and the release of the pro-inflammatory mediator nitric oxide (NO) was investigated (Figure 2). Results indicated that incubation with milk reduced the release of both cytokines (IL-18, IL-1β, and TNF-α) and NO induced by IL-6 (*p* < 0.05) (Figure 2 and Figure S2). The beneficial effects (*p* < 0.05) of Hol TMR and Hol P showed no differences related to feeding system. A more consistent decreasing effect on cytokine release was reached with Mod P (*p* < 0.01 vs. IL-6), as assessed by ELISA assays and immunoblotting analyses (Figure 2 and Figure S2). Similarly, Mod P treatment resulted as the most effective in attenuating the NO release induced by IL-6 incubation (*p* < 0.01 vs. IL-6) (Figure 2).

### 2.5. Milk Extract Effects on Oxidative Stress

**Figure 3:**
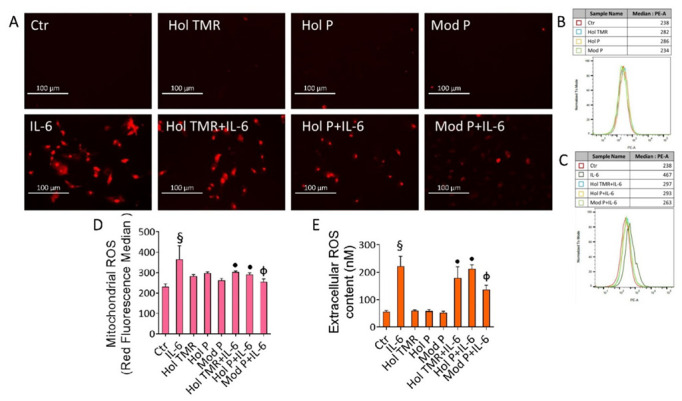
Milk extract effects on oxidative status. (**A**) Representative images by fluorescence microscopy and (**B**–**D**) FACS analysis, expressed as fluorescence median ± SD of n = 3 independent experiments, of mitochondrial and (**E**) extracellular ROS detection in ECs treated with Hol TMR, Hol P, and Mod P milk (20% *v*/*v*) for 72 h, IL-6 (20 ng/mL) for 24 h, or pretreated for 16 h with milk before stimulation for 24 h with IL-6. Scale bars = 100 µm. Control cells (Ctr) were treated with the corresponding highest volume of HBSS-10 mM Hepes: § *p* < 0.001 vs. Ctr; • *p* < 0.05 vs. IL-6; Φ *p* < 0.01 vs. IL-6.

We next evaluated the capacity of milk as a modulator of oxidative stress. In detail, results showed the absence of extracellular and mitochondrial ROS induction following incubation with milk. IL-6-mediated inflammatory stress provoked in ECs a massive mitochondrial ROS generation, related to the mitochondrial damage and intrinsic apoptotic cell death (*p* < 0.001) (Figure 3). This cellular event was also accompanied by exacerbating extracellular ROS release, known to be implicated in the pathogenesis of inflammatory-mediated diseases, as revealed by the cell impermeable Amplex red probe, able to specifically detect the extracellular ROS amount (*p* < 0.001) (Figure 3). Of interest, pre-incubation with Hol TMR and Hol P prevented the IL-6-induced ROS generation (*p* < 0.05), with the most prominent effect elicited by Mod P (*p* < 0.01) (Figure 3). The ROS inducer menadione was tested as a positive control (Figure S3).

### 2.6. Milk Extract Effects on Cell Death Mechanisms

**Figure 4:**
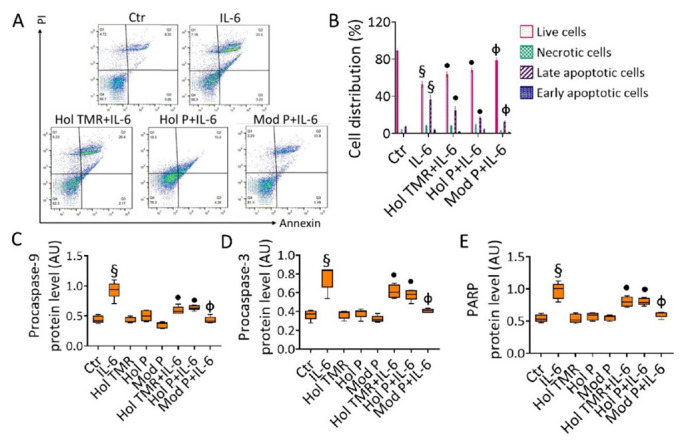
Milk extract effects on cell death. (**A**) Representative dot plots and (**B**) FACS analyses of Annexin V-FITC and Propidium Iodide (PI)-stained ECs treated with Hol TMR, Hol P, and Mod P milk (20% *v*/*v*) for 72 h, IL-6 (20 ng/mL) for 24 h, or pretreated for 16 h with milk before starting stimulation for 24 h with IL-6. Control cells (Ctr) were treated with the corresponding highest volume of HBSS-10 mM Hepes. Western blotting analysis of (**C**) procaspase-9, (**D**) procaspase-3, and (**E**) PARP expression levels represented as boxplots of densitometric mean values, expressed as arbitrary units (AU) of n = 5 independent experiments: § *p* < 0.001 vs. Ctr; • *p* < 0.05 vs. IL-6; Φ *p* < 0.01 vs. IL-6.

Treatment with IL-6 determined a decrease in viable cells (56.3% ± 3.11 vs. 86.7% ± 2.48 of Ctr, *p* < 0.001) with a concomitant increase in apoptotic cells (33.5% ± 3.06 vs. 8.32% ± 1.59 of Ctr, *p* < 0.001) (Figure 4A,B). Preventive incubation with Hol TMR and Hol P counteracted such toxic effect on viable (62.3% ± 1.55 and 70.3% ± 4.03 for Hol TMR and P, respectively) and apoptotic cells (28.6% ± 2.77 and 19.7% ± 5.11 for Hol TMR and P, respectively), displaying similar effects independently from the two feeding systems (*p* < 0.05 vs. IL-6). Incubation with Mod P milk led to the highest action against the IL-6-induced apoptosis (81.4% ± 1.44 of live cells and 13.9% ± 1.79 of late apoptotic cells, *p* < 0.01) (Figure 4A,B). ECs that underwent pre-incubation with Hol TMR and Hol P also restored procaspase-9, procaspase-3, and poly (ADP-ribose) polymerase (PARP) expression levels (*p* < 0.05 vs. IL-6), even more when Mod P milk pre-treatment was performed (*p* < 0.01 vs. IL-6) (Figure 4C–E and Figure S4). As for viability assay, no apoptotic effects were reported when ECs were treated up to 72 h with milk (Figure S4).

### 2.7. Milk Extract Effects on Autophagy Induction

**Figure 5:**
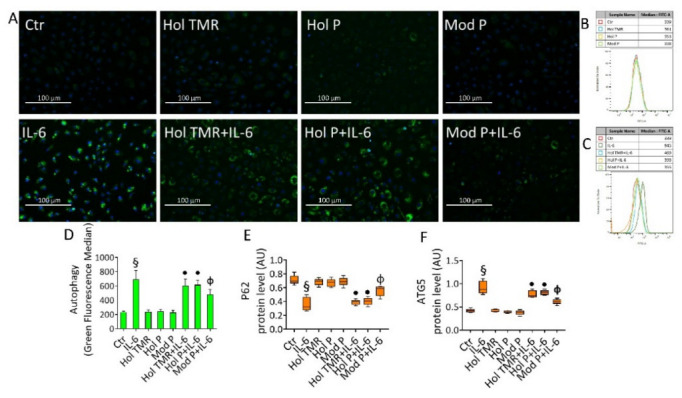
Milk extract effects on autophagy. (**A**) Representative images by fluorescence microscopy and (**B**–**D**) flow cytometry analysis, expressed as fluorescence median ± SD of n = 3 experiments, of green detection reagent in ECs treated with Hol TMR, Hol P, and Mod P milk (20% *v*/*v*) for 72 h, IL-6 (20 ng/mL) for 24 h, or pretreated for 16 h with milk before starting stimulation for 24 h with IL-6. Scale bars = 100 µm. Control cells (Ctr) were treated with the corresponding highest volume of HBSS-10 mM Hepes. Western blotting analysis of (**E**) p62 and (**F**) ATG5 expression levels represented as boxplots of densitometric mean values, expressed as arbitrary units (AU) of n = 5 different experiments: § *p* < 0.001 vs. Ctr; • *p* < 0.05 vs. IL-6; Φ *p* < 0.01 vs. IL-6.

IL-6- induced autophagy (*p* < 0.001 vs. Ctr) was inhibited by incubation with the milk, as evidenced by fluorescence microscopy and FACS analysis (Figure 5). Specifically, Hol TMR and Hol P were able to block the IL-6-induced autophagy (*p* < 0.05), but higher beneficial effects were elicited by Mod P (*p* < 0.01 vs. IL-6) (Figure 5A–D). Immunoblotting analysis of autophagic proteins showed the capability of milk to modulate the expression levels of p62 and ATG5 (*p* < 0.05 vs. IL-6, for Hol TMR and Hol P), with a higher effect exerted by Mod P (*p* < 0.01 vs. IL-6) (Figure 5E,F and Figure S5). The autophagy inducer rapamycin (1 µM overnight) was tested as a positive control (Figure S5).

## 3. Discussion

The results of this study showed an influence of breed in affecting the biomolecule content and the antioxidant activity of milk and dairy products, resulting in milk with functional proprieties able to prevent EC inflammatory damage induced by IL-6. Indeed, milk and dairy products from *Modicana*, with rustic and resistant features, were richer in bioactive compounds compared to *Holstein*. A previous work by Servillo et al. [9] showed the occurrence of health-promoting compounds with quaternary ammonium (betaine, l-carnitine, and short-chain acyl-carnitines) in the milk of different species and found a high variability. In particular, buffalo milk showed δ-valerobetaine, γ-butyrobetaine, glycine betaine, and propionyl-l-carnitine values three times higher than bovine, while l-carnitine and acetyl-l-carnitine showed values two-fold higher compared to both *Modicana* and *Holstein* breeds [20]. Moreover, treatment with milk reduced the IL-6-induced cytotoxicity, with *Modicana* milk exerting the most prominent protective effect against the release of inflammatory cytokines, ROS accumulation, and activation of programmed cell death pathways. In agreement with recent evidence [27,28], our in vitro studies suggest that milk, as part of an equilibrating dietary pattern, can play a role as a determinant or suppressor of low-grade chronic inflammation. In this regard, different ROS species trigger several signaling pathways, thus leading to different outcomes depending on their generation and subcellular localization. Chronic low-grade inflammation and oxidative stress are significant factors predisposing to insulin resistance, hypertension, and T2D. At the cellular level, increased levels of inflammatory markers, lipid peroxides, and free radicals result in cell damage and programmed cell death. Among the various long-term systemic effects, low-grade inflammation may affect insulin sensitivity, leading to metabolism impairment and increased risk of other diseases [29]. Chronic inflammation, in turn, can establish a vicious circle characterized by increased vascular inflammation, exacerbated autophagy, and apoptosis, culminating in impairment of endothelial function with cardiovascular systemic sequelae [30]. High levels of autophagy are associated with programmed cell death mechanisms such as apoptosis, ferroptosis, and necroptosis [12,15]. In this scenario, we investigated the effect of milk in the apoptotic and autophagic mechanisms in ECs under IL-6-mediated inflammatory stress. We showed that IL-6 induced autophagic cell death and apoptosis and that milk treatment was able to oppose both programmed phenomena, with most prominent effects exerted by Mod P extract incubation.

To date, pure bioactive compounds identified in the milk, such as δ-valerobetaine and γ-butyrobetaine, along with their synergistic effects, have been tested by using different in vitro cell models, showing anticancer effects in head and neck squamous cell carcinoma Cal 27 [13] and in HT-29 and LoVo colon cancer cells [12]. In ECs, pure δ-valerobetaine protects against oxidative stress and cytokine release during short-term treatment with high levels of glucose [31]. Additionally, recent data showed that δ-valerobetaine also protected ECs against PA-induced insulin resistance (IR) by limiting the inhibitory phosphorylation of IRS1 and restoring phosphorylated Akt expression levels. In particular, δ-valerobetaine reduced oxidative stress and cytokine levels, restored the expression levels of mitochondrial sirtuin 3, and attenuated pyroptosis and autophagy [32]. Further studies are undoubtedly necessary for a deeper understanding of biological effects caused by bioactive compounds identified in the milk and/or their mixtures in the prevention of EC inflammation.

The bioactive compounds, as well as betaines and short-chain acylcarnitines, occurring in 3 KDa milk samples used in this study, displayed anti-inflammatory properties. These water-soluble compounds, potentially produced endogenously, are related to the dietary intake of certain foods such as whole grains and milk and resulted as metabolically important [33]. Metabolic disorders due to dysfunctional nutrition represent a health problem related to obesity and its morbidities [27]. Given the causal link between obesity and systemic inflammation [28], we chose the in vitro model of endothelial cells to generate new knowledge on the metabolic role of the bioactive compounds of milk. The feeding system, in our study limited to *Holstein*, influenced biomolecule compounds only in the milk and whey without modifying antioxidant power. Indeed, the TMR characterized by maize silage, hay, and concentrate improved the concentration of some health-promoting biomolecules in milk and whey compared to the pasture-based feeding system. In this context, concentrate could contribute to improve the trimethyllysine (TML) content, the main precursor of carnitine [34]. However, the carnitine concentration in animals could be influenced by several factors, including the animal microbiota, the carnitine endogenous synthesis, absorption from the gastrointestinal tract, and the excretion [35]. l-carnitine not obtained from food is synthesized endogenously from two essential amino acids, lysine and methionine, in a multi-step process occurring across several cell compartments (cytosol, lysosomes, and mitochondria) [36]. In previous studies, buffaloes fed TMR with an inclusion of 30% green forage received 25 kg of alfalfa [20], known to contain from 2 to 10 mg/kg of TML [34]. Here, *Holstein* fed TMR received concentrate, maize silage, and soybean, having a higher TML concentration [34]. This could explain the higher concentration of biomolecules in milk and in whey compared to *Holstein* fed on natural polyphite pasture with poor TML amount.

It is worth underlining that the results reported herein should be considered in the light of one major limitation, which is the lack of comparison of physico-chemical and microbiological indicators of milk (i.e., pH, density, mineral salt indicators).

Nonetheless, the results of this study suggest the importance of the inclusion of TML precursors in the feeding system in order to positively modulate the bioactive compounds in milk and dairy products.

In conclusion, taken together, the results of this study suggest that breed and feeding system can impact the milk content of bioactive compounds, implementing the bioactivity of milk and making it a potentially effective food in the maintenance of endothelial redox homeostasis during inflammation.

## 4. Materials and Methods

### 4.1. Experimental Design

In order to understand if breed and feeding system could have a role in modulating (i) health-promoting biomolecules’ content, (ii) the antioxidant activity of milk and dairy products, and (iii) the inflammatory properties of milk, two different experimental designs were performed. The first one was carried out in order to see the role of breed in modulating biomolecules’ content and antioxidant activity of milk and dairy products, and two different bovine breeds were chosen (*Modicana* vs. *Holstein*). At the same time, limited to *Holstein*, the second experimental design was aimed to understand whether the modulation of the feeding system could improve the bioactive profile of milk and dairy products. Hence, two different feeding systems were used (pasture vs. TMR). The latter experiment was performed only in *Holstein*, since the *Modicana* is usually bred extensively. All the milk of the animals produced during the two experimental designs was used to assess its ability to prevent EC inflammatory stress induced by IL-6.

### 4.2. Experimental Design 1—Influence of Breed on the Functional Molecules and Antioxidant Activity of Milk and Dairy Products

The study was carried out in four dairy farms, two for *Holstein* (Hol) breed and two for *Modicana* (Mod) breed, all of them located in Ragusa Province (RG). Among the two *Holstein* farms (group Hol P; n = 30), animals were fed on natural polyphite pasture all day with the integration of concentrate (about 8 kg), hay (about 4 kg), and maize silage (about 15 kg) according to changes in pasture compositions. The composition of the pasture was typical of this area [37] and diet was characterized on the dry matter (DM) basis by 14% crude protein, 18% starch, 37% neutral detergent fiber, 37% non-structural carbohydrate, and 0.90 MFU (Milk Forage Units = 1700 Kcal). Among the two *Modicana* farms (group Mod P; n = 30), animals were fed polyphite meadow pasture all day with the integration of concentrate (about 6.3 kg) and hay (about 6 kg) according to changes in pasture compositions. Diet chemical composition was characterized on DM basis: 12.6% crude protein, 6% starch, 37.9% neutral detergent fiber, 40% non-structural carbohydrate, 0.80 MFU.

Feedstuff and pasture were sampled and analyzed monthly according to AOAC [38]. Energy values (MFU = 1700 kcal) were calculated using equations provided by INRA [39]. Diet supplementation was modified considering the chemical pasture composition to guarantee animals’ requirements.

The trial lasted five months. Animals were on pasture all day and milked twice a day, manually for *Modicana* and in the milking parlor for *Holstein*. Animals were selected and divided into the experimental groups according to days in milk, parity (only pluriparous were enrolled in the experiment), and milk yield, recorded once two weeks before the start of the trial.

### 4.3. Experimental Design 2—Influence of Feeding System on the Biomolecule Content and Antioxidant Activity of Milk and Dairy Products

The study was carried out in four *Holstein* dairy farms, all of them located in Ragusa Province (RG). Among the four *Holstein* farms, two (group Hol TMR; n = 30) were fed a total mixed ratio (TMR) characterized by maize silage (16 kg), hay (7 kg), and concentrate (10 kg). Diet chemical composition was characterized on DM basis by 15% crude protein, 23% starch, 36% neutral detergent fiber, 38% non-structural carbohydrate, and 0.91 MFU. The other two farms (group Hol P; n = 30) were the same as the first experimental design and fed animals on natural polyphite pasture, as reported in the experimental design 1 (group Hol P; n = 30).

Feedstuff and pasture were sampled and analyzed monthly according to AOAC [38]. Energy values (MFU = 1700 kcal) were calculated using equations provided by INRA [39]. Diet supplementation was modified considering the chemical pasture composition to guarantee animals’ requirements. The trial lasted five months. The animals fed TMR were maintained in pens with a concrete floor and were fed and milked twice daily in the morning and afternoon in the milking parlor. Animals were selected and divided into the experimental groups according to days in milk, parity (only pluriparous were enrolled in the experiment), and milk yield, recorded once two weeks before the start of the trial.

### 4.4. Milk and Dairy Product Sampling and Preparation

Bulk milk samples from animals belonging to the two experimental designs were collected once a month for five months. On the same days, samples of whey, ricotta, and mozzarella cheese from each experimental group (experimental designs 1 and 2) were collected. Aliquots (2 mL) of milk or whey were first skimmed by centrifugation at 3000× *g* for 15 min a 4 °C to remove the fat globules. The skimmed milk was filtered through 5 μm Millipore filters (Millipore, Bedford, MA, USA) and then stored frozen in aliquots until used. Ricotta and mozzarella cheese (1 g), cut into small pieces, were first homogenized in an ice chamber with three parts of 0.1% (*w*/*w*) formic acid precooled at 4 °C and then centrifuged in a Precellys 24 system (Bertin Technologies, Rockville, MD, USA) at 800× *g* for 10 min at 4 °C to collect the supernatant. The supernatant was again centrifuged at 10,000× *g* at 4 °C for 10 min and finally filtered sequentially through 5 μm and 0.45 μm Millipore filters. All extracts were prepared in triplicate and then stored frozen in aliquots until used. Before mass spectrometric analysis, all specimen aliquots were filtered through Amicon Ultra 0.5 mL centrifugal filters (Millipore, Bedford, MA, USA) with 3 kDa molecular weight cutoff.

### 4.5. Betaine and Carnitine Profile by HPLC-ESI-MS/MS Analysis

Extracts of milk, whey, ricotta, and mozzarella cheese produced by the bulk milk of the different farms involved for both experimental designs were prepared and analyzed by HPLC electrospray ionization-tandem MS for l-carnitine, acetyl-l-carnitine, propionyl-l-carnitine, glycine betaine, γ-butyrobetaine, and δ-valerobetaine, as described by Servillo et al. [9,16].

Briefly, the chromatography was conducted isocratically with 0.1% formic acid in water at a flow rate of 100 μL/min. Volumes of 10 µL of standard solution or sample were injected. Compounds were identified based on their retention times and MS2 fragmentation patters. The concentration of each substance was determined by comparison of the peak area of its most intense MS2 fragment with the respective calibration curve built with standard solutions. HPLC-ESI-MS/MS analyses were conducted with an Agilent LC-MSD SL quadrupole ion trap, in positive multiple reaction monitoring (MRM) using for each analyte the most intense MS2 transitions. The linear regression analysis was achieved by plotting the peak areas of the monitored fragment ions versus the concentrations of the analyte standard solutions. Each sample was analyzed in triplicate and the mean concentration value of each compound was expressed in mg/kg for ricotta and mozzarella cheese or mg/L for milk and whey. Linearity was assessed by correlation coefficients (r^2^) > 0.99 for all compounds. Precision and accuracy for all compounds in samples ranged from 95% to 105%. Coefficients of variations ranged between 8.47 and 12.95 for betaine and 7.76 and 10.36 for carnitine in both milk and dairy products.

### 4.6. FRAP and TAC Assay

For both experimental designs, the total antioxidant capacity (TAC) was assessed using a colorimetric assay (ab65329, Abcam, Cambridge, UK) based on the Cu^2+^ conversion to Cu^+^ by antioxidants and to the following release of a colorimetric probe, proportional to the total antioxidant power, following the manufacturer’s instruction. Briefly, 3 kDa samples were mixed with 100 µL of the Cu^2+^ working solution and then incubated for 90 min at room temperature in the dark. The reaction was detected by measuring the absorbance at 570 nm with microplate reader model 680 Bio-Rad (Bio-Rad, Hercules, CA, USA). Absorbance was interpolated with the standard curve of Trolox, a known antioxidant, and the total antioxidant capacity expressed as nM equivalents. The ability of samples to reduce ferric iron (Fe^3+^) to ferrous iron (Fe^2+^) by generating a colorimetric reaction was carried out using the ferric reducing antioxidant power assay (FRAP) (MAK369, Sigma Aldrich, St. Louis, MO, USA). The reducing power of the samples was calculated by reacting 10 μL of sample with 190 μL of the reaction mixture and monitoring the increase in absorbance at 594 nm for 1 h at 37 °C. The reducing proprieties of samples was determined using a ferrous iron standard curve and results are expressed as Fe^2+^ equivalents (nM).

### 4.7. Cell Culture and Treatments

The human aorta endothelial cells, ECs (TeloHAEC cell line, CRL-4052), were purchased from American Type Culture Collection (ATCC, Manassas, VA, USA) and grown as a monolayer in Vascular Cell Basal Medium (PCS-100-030, ATCC, Manassas, VA, USA) supplemented with Endothelial Cell Growth kit-VEGF (PCS-100-041, ATCC, Manassas, VA, USA), at 37 °C under a humidified atmosphere with 5% CO_2_. For EC treatment, milk samples (2 mL) were first skimmed by centrifugation at 3000× *g* for 15 min at 4 °C to remove the fat globules. The supernatant was centrifuged at 10,000× *g* at 4 °C for 10 min and then filtered through Amicon Ultra 0.5 mL centrifugal filters (Millipore, Bedford, MA, USA) with 3 kDa molecular weight cutoff. Before endothelial cell incubation, 3 KDa milk specimens were filtered with 0.22 μm Millipore filters (Millipore, Bedford, MA, USA). EC treatment was performed by using 3 KDa milk samples, as previously reported [27]. In detail, ECs were incubated for 24, 48, and 72 h with increasing volumes (5, 10, 15, 20 µL) of Hol TMR, Hol P, and Mod P milk in complete culture medium. To mimic inflammatory condition, human interleukin-6 (IL-6), dissolved in Hanks’ balanced salt solution (HBSS)-10 mM Hepes at 100 μg/mL stock concentration in sterile phosphate buffered saline (PBS) containing 1% bovine serum albumin, was used. ECs were treated with IL-6 up to 20 ng/mL and incubated for 6, 12, 18, and 24 h. In order to provide the cytoprotective effects of milk, ECs were pre-incubated for 16 h with milk, before starting IL-6 stimulation for 24 h. Control cells (Ctr) were maintained in complete culture medium with the corresponding highest volume of HBSS 10 mM Hepes.

### 4.8. Cell Viability Detection

For cell-based treatment, the incubation time of 72 h with 3 KDa milk extracts was chosen to first provide the effect of milk on EC viability, as previously reported [31]. Cultured cells, indeed, in drug-like compound dose–response assays, including CCK-8 assay, are typically exposed to treatment up to 72 h. Particularly, EC viability was detected by Cell Counting Kit-8 (CCK-8, Donjindo Molecular Technologies, Inc., Rockville, MD, USA), according to manufacturer’s instructions. Briefly, CCK-8 solution (10 μL) was added to each well and then plate incubated for 4 h at 37 °C. Cell absorbance was measured at 450 nm with microplate reader (model 680, Bio-Rad, Hercules, CA, USA) and viability expressed both as percentage of control and as the mean of the optical density at 450 nm ± Standard Deviation (SD). All experiments were performed with n = 5 replicates.

### 4.9. Cytokine Level Determination

Levels of cytokines (IL-18, IL-1β, and TNF-α) were determined by ELISA assays (human interleukin-18 ELISA, RAF143R, BioVendor Laboratorni medicina a.s., Brno, Czech Republic; human IL-1β quantikine ELISA, DLB50, R&D Systems, Inc. Minneapolis, MN, USA; ELISA Cymax TNF-alpha ELISA, YIF-LF-EK0193, AbFrontier, Seoul, Korea, respectively), according to the manufacturer’s protocols. EC supernatants (100 μL) were incubated for 1 or 2 h in microplate wells precoated with specific anti-cytokine antibodies. After 1 h incubation and washing to remove non-bound cytokines, a biotin-labeled antibody for each cytokine was added and incubated for an additional hour, followed by a 30 min incubation with streptavidin-HRP conjugate antibody. The absorbance was measured at 450 nm using a microplate reader (model 680, Bio-Rad, Hercules, CA, USA) and cytokine levels in samples determined by plotting the absorbance values with each standard curve.

### 4.10. Nitric Oxide Level Assessment

The content of nitric oxide was detected by the colorimetric nitric oxide assay kit (ab65328, Abcam, Cambridge, UK), following indication of the manufacturer. Then, 5 μL of deproteinated sample extracts, obtained from 2 × 10^6^ cells, were added to each well and incubated at room temperature for 1 h. Thereafter, enhancer was added and incubated at room temperature for a further 10 min before adding Griess reagents to measure absorbance with a microplate reader (model 680, Bio-Rad, Hercules, CA, USA). Total nitric oxide content was determined by plotting sample values to standard curve and expressed as µmol/millions of cells.

### 4.11. Oxidative Stress Evaluation

Mitochondrial ROS content was assessed with MitoSOX Red Mitochondrial Superoxide Indicator (M36008, Invitrogen, Waltham, MA, USA), according to manufacturer’s indications. After treatments, ECs were stained for 30 min with 5 µM MitoSOX fluorogenic probe in complete medium. Cells were imaged on a fluorescence microscope EVOS FL Cell Imaging System (Thermo Scientific, Rockford, IL, USA) and then mitochondrial ROS fluorescence intensities quantified using a BD Accuri C6 cytometer (BD Biosciences, San José, CA, USA). At least 20,000 events were recorded for each sample and analysis performed with FLOWJO V10 software (Williamson Way, Ashland, OR, USA). Extracellular ROS amount was assessed with Amplex Red Hydrogen Peroxide/Peroxidase Assay kit (A22188, Invitrogen, Waltham, Ma, USA). Amplex red reagent (100 μL), containing 50 μM Amplex Red and 0.1 U HRP/mL, was added to an EC suspension (20 μL), containing 2 × 104 live cells, in Krebs-Ringer phosphate glucose buffer. After 1 h incubation at 37 °C, fluorescence of the oxidized product, 10-acetyl-3,7-dihydroxyphenoxazine, was measured with a multiplate reader (model Infinite 2000, Tecan, Männedorf, Switzerland) using 530 nm excitation wavelength and 590 nm emission wavelength. H_2_O_2_ extracellular content was quantified using a standard curve (0–2 μM concentration range). Experiments were performed with n = 5 replicates. The ROS inducer menadione (50 µM) (M57405, Sigma Aldrich, St. Louis, MO, USA) was incubated for 30 min at 37 °C and used as positive control.

### 4.12. Apoptosis Detection

In order to distinguish apoptotic and necrotic from live cells, the FITC Annexin V Apoptosis detection kit (556547, BD Pharmigen, Franklin Lakes, NJ, USA) was used. After treatments, cells were detached by trypsinization and washed twice with PBS, resuspended in 500 μL binding buffer 1×, and incubated with 2 μL Annexin V-FITC and 2 μL of the vital dye Propidium Iodide, PI (20 μg/mL), for 30 min. Detection of viable, necrotic, early, and late apoptotic cells was performed using a BD Accuri™ C6 (BD Biosciences) cytometer and data analyzed by FlowJo V10 software (Williamson Way, Ashland, OR, USA). For each sample, 20,000 events were recorded.

### 4.13. Autophagy Assessment

Autophagy was detected by labeling, for 30 min at 37 °C in the dark, and ECs were treated with Autophagy Assay Kit (ab139484, Abcam), following the manufacturer’s indication. Cells were then washed with PBS and imaged on a fluorescence microscope EVOS FL Cell Imaging System (Thermo Scientific, Rockford, IL, USA), before FACS analysis. For each sample at least 20,000 events were recorded.

### 4.14. Cell Lysis and Immunoblotting Analysis

ECs were lysed in lysis buffer (1% NP-40, 0.5% sodium deoxycholate, 0.1% SDS in PBS) containing 10 μg/mL aprotinin, leupeptin, and 1 mM phenylmethylsulfonyl fluoride. Extracted proteins (30–60 μg) were separated by sodium dodecyl sulfate-polyacrylamide gel electrophoresis (SDS-PAGE) and then transferred to nitrocellulose membrane (Bio-Rad). Membranes were incubated for 1 h at room temperature (RT) with 1× TBS 1% casein blocker (1610782, Bio-Rad, Bio-Rad, Hercules, CA, USA) under gentle shaker. Membranes were then incubated at 4 °C overnight with primary antibodies: anti-tumor necrosis factor alpha (TNF-α, 1:1000, ab6671, Abcam, Abcam, Cambridge, UK); anti-IL-1β (1:1000, ab216995, Abcam, Cambridge, UK); anti-IL-18 (1:1000, ab243091, Abcam, Cambridge, UK); anti-autophagy-related 5 (ATG5, 1:1000, 9980, Cell Signaling Technology, Danvers, MA, USA); anti-sequestosome-1 (SQSTM1/p62, 1:2000, 5114, Cell Signaling Technology, Danvers, MA, USA); anti-caspase-3 (1:1000, 9662, Cell Signaling Technology, Danvers, MA, USA); anti-caspase-9 (1:1000, 9508, Cell Signaling Technology, Danvers, MA, USA); anti-poly(ADP ribose) polymerase (PARP, 1:1000, ab194586, Abcam, Cambridge, UK); anti-α-tubulin (1:5000, E-AB-20036, Elabscience Biotechnology Inc., Houston, TX, USA); anti-actin (1:3000, ab179467, Abcam, Cambridge, UK), and anti-glyceraldehyde-3-phosphate dehydrogenase (GAPDH, 1:2000, ab9485, Abcam, Cambridge, UK). Secondary HRP-conjugated anti-mouse or anti-rabbit antibodies were incubated for 1 h at room temperature and immunocomplexes revealed with Excellent Chemiluminescent Substrate kit (E-IR-R301, Elabscience Biotechnology Inc., Houston, TX, USA). The densities of immunoreactive bands, acquired by using ChemiDoc Imaging System with Image Lab 6.0.1 software (Bio-Rad Laboratories, Milan, Italy), were measured by ImageJ software 1.52n version (Wayne Rasband, National Institutes of Health, Bethesda, MD, USA) and expressed as arbitrary units (AU).

### 4.15. Statistical Analyses

Statistical analyses were performed using SPSS (23.0) for Windows 10 (SPSS Inc., Chicago, IL, USA) [40]. To compare data for milk yield, TAC, FRAP, and health-promoting biomolecules of milk and dairy products, ANOVA for repeated measure (scaled identity was the covariance structure) was used. The pairwise contrasts were performed with breed as fixed effect for the experimental design 1 (*Holstein* and *Modicana* breeds fed pasture) and diet for experimental design 2 (*Holstein* cows fed TMR and pasture). Results are mean ± standard error mean (sem). A statistically significant difference was accepted at *p* < 0.05. For in vitro studies on EC, analyses were performed and graphs created using GraphPad Prism version 9.1.2 (GraphPad Software Inc., San Diego, CA, USA).

## 5. Conclusions

The use of pasture improves the quality and cytoprotective effects of milk and contributes to the persistence and development of autochthonous breeds such as *Modicana*. Undoubtedly, compared to milk, the proportional content of functional biomolecules in dairy products is lower. However, their intake, as part of a balanced human diet including milk, can contribute to the maintenance of health and well-being of consumers. The research in the functional food field is ongoing and far from conclusive. It is certainly difficult to identify the cause–effect relationships triggered by the 3 kDa fraction of milk used in this study due to the presence of multiple functional components, including small peptides, capable of interacting with each other even in a synergistic way.

In this scenario, the involvement of the gut microbiota, the immune system, and the epigenetic pathways through which milk affects the system’s homeostasis of endothelial cells could provide a glimpse of how extraordinarily complex the effect of milk on human health is, far beyond the most obvious vascular outcomes. The aforementioned beneficial effect of the 3 kDa milk fraction, able to counteract in vitro the deleterious effects of the chronic inflammation state, may lead consumers to consider milk as a source of specific nutrients with health-promoting properties. Furthermore, consumer awareness of the origin of milk from animals fed in a natural way can favor its consumption and, as regards the *Modicana* breed, helps to preserve this breed which plays an important social, economic, and environmental role.

## Figures and Tables

**Table 1:** Effect of breed on health-promoting biomolecules in milk and dairy products. The pairwise contrast involved *Holstein* (Hol) and *Modicana* (Mod) breeds fed pasture (P).

		γ-Butyrobetaine	δ-Valerobetaine	Glycine Betaine	l-Carnitine	Acetyl-l-Carnitine	Propionyl-l-Carnitine
**Milk (mg/L)**	**Hol P**	1.73 ± 0.08 ^X^	7.44 ± 0.12 ^X^	6.76 ± 0.09 ^X^	17.53 ± 0.24 ^X^	19.92 ± 0.35 ^x^	7.12 ± 0.13 ^X^
	**Mod P**	2.18 ± 0.06 ^Y^	7.89 ± 0.09 ^Y^	7.11 ± 0.10 ^Y^	18.80 ± 0.24 ^Y^	20.91 ± 0.22 ^y^	7.85 ± 0.11 ^Y^
**Whey (mg/Kg)**	**Hol P**	1.25 ± 0.06 ^X^	6.02 ± 0.22 ^X^	5.81 ± 0.10 ^X^	15.47 ± 0.30 ^x^	15.21 ± 0.15 ^x^	5.86 ± 0.17
	**Mod P**	1.61 ± 0.05 ^Y^	6.84 ± 0.17 ^Y^	6.23 ± 0.10 ^Y^	16.49 ± 0.26 ^y^	15.75 ± 0.17 ^y^	6.13 ± 0.11
**Ricotta cheese (mg/Kg)**	**Hol P**	1.19 ± 0.65 ^x^	5.59 ± 0.25	5.69 ± 0.19	14.80 ± 0.43	14.43 ± 0.16 ^x^	5.48 ± 0.09 ^x^
	**Mod P**	1.39 ± 0.48 ^y^	5.86 ± 0.20	5.82 ± 0.17	15.20 ± 0.33	14.98 ± 0.14 ^y^	5.88 ± 0.13 ^y^
**Mozzarella cheese (mg/Kg)**	**Hol P**	1.25 ± 0.04 ^X^	6.24 ± 0.30	5.88 ± 0.19	15.37 ± 0.36	15.53 ± 0.17	6.51 ± 0.15
	**Mod P**	1.51 ± 0.04 ^Y^	6.38 ± 0.22	6.00 ± 0.14	15.87 ± 0.25	15.83 ± 0.14	6.33 ± 0.09

^X^, ^Y^, Values with different superscript are significantly different; *p* < 0.01; ^x^, ^y^, values with different superscript are significantly different; *p* < 0.05.

**Table 2:** Effect of breed on antioxidant activity in milk and dairy products. The pairwise contrast involved *Holstein* (Hol) and *Modicana* (Mod) breeds fed pasture (P).

		TAC	FRAP
**Milk (nmol/L)**	**Hol P**	87.48 ± 2.81 ^X^	104.94 ± 9.02 ^X^
	**Mod P**	112.46 ± 3.25 ^Y^	144.97 ± 6.13 ^Y^
**Whey (nmol/Kg)**	**Hol P**	63.97 ± 3.22 ^X^	53.88 ± 3.10 ^X^
	**Mod P**	89.92 ± 1.87 ^Y^	72.66 ± 1.69 ^Y^
**Ricotta cheese (nmol/Kg)**	**Hol P**	42.31 ± 1.84 ^X^	33.97 ± 1.59 ^X^
	**Mod P**	55.34 ± 1.65 ^Y^	47.85 ± 1.96 ^Y^
**Mozzarella cheese (nmol/Kg)**	**Hol P**	59.82 ± 2.53 ^X^	50.34 ± 2.28 ^X^
	**Mod P**	77.08 ± 1.75 ^Y^	66.71 ± 1.67 ^Y^

FRAP = ferric reducing antioxidant power assay; TAC = total antioxidant capacity; ^X^, ^Y^, values with different superscript are significantly different; *p* < 0.01.

**Table 3:** Effect of feeding system on health-promoting biomolecules in milk and dairy products. The pairwise contrast involved *Holstein* (Hol) fed total mixed ratio (TMR) and pasture (P).

		γ-Butyrobetaine	δ-Valerobetaine	Glycine Betaine	l-Carnitine	Acetyl-l-Carnitine	Propionyl-l-Carnitine
**Milk (mg/L)**	**Hol TMR**	2.08 ± 0.11 ^X^	7.87 ± 0.19 ^X^	6.94 ± 0.15	18.79 ± 0.43 ^x^	20.45 ± 0.27	7.16 ± 0.18
	**Hol P**	1.73 ± 0.08 ^Y^	7.44 ± 0.12 ^Y^	6.76 ± 0.09	17.53 ± 0.24 ^y^	19.92 ± 0.35	7.12 ± 0.13
**Whey (mg/Kg)**	**Hol TMR**	1.42 ± 0.11	7.05 ± 0.35 ^X^	6.52 ± 0.19 ^X^	17.68 ± 0.55 ^X^	16.47 ± 0.28 ^X^	6.06 ± 0.13
	**Hol P**	1.25 ± 0.06	6.02 ± 0.22 ^Y^	5.81 ± 0.10 ^Y^	15.47 ± 0.30 ^Y^	15.21 ± 0.15 ^Y^	5.86 ± 0.17
**Ricotta cheese (mg/Kg)**	**Hol TMR**	1.22 ± 0.07	5.63 ± 0.26	5.43 ± 0.15	14.44 ± 0.43	14.20 ± 0.20	5.48 ± 0.19
	**Hol P**	1.19 ± 0.65	5.59 ± 0.25	5.69 ± 0.19	14.80 ± 0.43	14.43 ± 0.16	5.89 ± 0.15
**Mozzarella cheese (mg/Kg)**	**Hol TMR**	1.43 ± 0.08	6.38 ± 0.26	6.04 ± 0.20	15.38 ± 0.42	15.37 ± 0.26	6.22 ± 0.12
	**Hol P**	1.25 ± 0.04	6.24 ± 0.30	5.88 ± 0.19	15.37 ± 0.36	15.53 ± 0.17	6.51 ± 0.15

^X^, ^Y^, Values with different superscript are significantly different; *p* < 0.01; ^x^, ^y^, values with different superscript are significantly different; *p* < 0.05.

**Table 4:** Effect of feeding system on antioxidant activity in milk and dairy products. The pairwise contrast involved *Holstein* (Hol) fed total mixed ratio (TMR) and pasture (P).

		TAC	FRAP
**Milk (nmol/L)**	**Hol TMR**	89.39 ± 3.79	94.34 ± 9.49
	**Hol P**	87.48 ± 2.81	104.94 ± 9.02
**Whey (nmol/Kg)**	**Hol TMR**	57.08 ± 4.93	47.70 ± 5.13
	**Hol P**	63.97 ± 3.22	53.88 ± 3.10
**Ricotta cheese (nmol/Kg)**	**Hol TMR**	41.43 ± 3.00	33.18 ± 2.83
	**Hol P**	42.31 ± 1.84	33.97 ± 1.59
**Mozzarella cheese (nmol/Kg)**	**Hol TMR**	59.28 ± 2.70	48.87 ± 2.64
	**Hol P**	59.82 ± 2.53	50.34 ± 2.28

FRAP = ferric reducing antioxidant power assay; TAC = total antioxidant capacity.

## Data Availability

Not applicable.

## Supplementary Materials

The following supporting information can be downloaded at: https://www.mdpi.com/article/10.3390/ijms231911088/s1.
