# Supplementary material for: Breed and Feeding System Impact the Bioactive Anti-Inflammatory Properties of Bovine Milk
Source: Int J Mol Sci. 2022 Sep 21;23(19):11088. doi: 10.3390/ijms231911088 (PMC9569879; doi:10.3390/ijms231911088)
Supplement: Supplementary file 1 [file ijms-23-11088-s001.zip › ijms-1910652-supplementary.pdf]

Breed and feeding system impact the bioactive anti-inflammatory properties of bovine milk

Angela Salzano [1,§], Maria Chiara Di Meo [2,§], Nunzia D'Onofrio [3], Giovanna Bifulco [1], Alessio Cotticelli [1,*], Francesca Licitra [4], Antonio Iraci Fuintino [5], Giuseppe Cascone [4], Maria Luisa Balestrieri [3], Ettore Varricchio [2] and Giuseppe Campanile [1]

[1] Department of Veterinary Medicine and Animal Productions, University of Naples Federico II, 80137 Naples, Italy
[2] Department of Science and Technology, University of Sannio, 82100 Benevento, Italy
[3] Department of Precision Medicine, University of Campania Luigi Vanvitelli, 80138 Naples, Italy
[4] Istituto Zooprofilattico Sperimentale della Sicilia "A. Mirri", 90129 Palermo, Italy
[5] Provincial Public Health Authority of Ragusa, 97100 Ragusa, Italy
§ The authors contributed equally to this work.
* Correspondence: alessio.cotticelli@unina.it

SUPPLEMENTARY MATERIALS

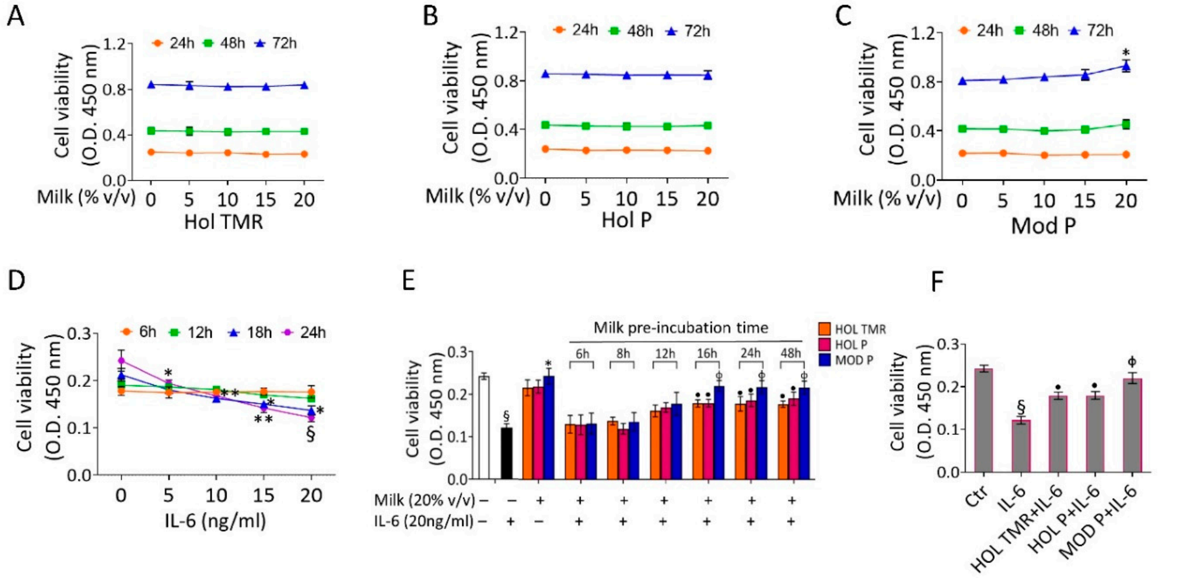

Figure S1. Milk effects on EC viability. EC viability after exposure to different volumes (0-20% v/v) of (A) Hol TMR, (B) Hol P, and (C) Mod P milk up to 72h or to (D) different concentrations (0-20 ng/ml) of IL-6 up to 24h. (E) Time-course experiments of milk pre-incubation times before starting inflammatory induction with IL-6 (20 ng/ml) and (F) EC pretreated for 16h with milk (20% v/v) before starting stimulation for 24h with IL-6 (20 ng/ml). Cell viability was assessed by Cell Counting Kit-8 assay (Donjindo Molecular Technologies) and reported as optical density (O.D.) values. Control cells (0 or Ctr) were treated with the corresponding highest volume of HBSS-10 mM Hepes. *$p < 0.05$ vs 0 mM or Ctr; **$p < 0.01$ vs 0 mM or Ctr; §$p < 0.001$ vs 0 mM or Ctr; •$p < 0.05$ vs IL-6; Φ$p < 0.01$ vs IL-6.

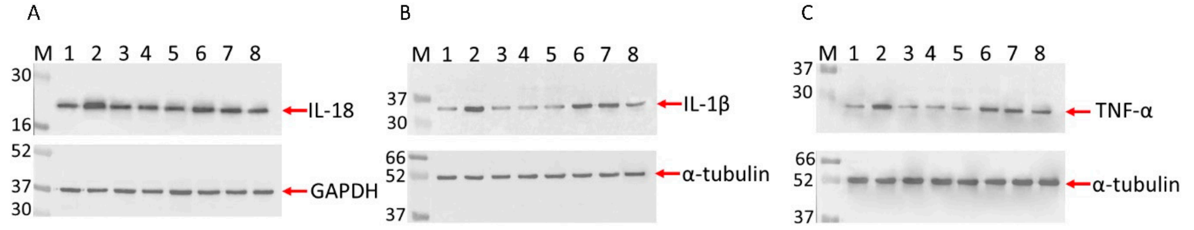

Figure S2. Cytokine levels. Representative cropped blots of (A) IL-18, (B) IL-1β and (C) TNF-α immunoblotting analysis in EC treated with Hol TMR, Hol P, and Mod P milk (20% v/v) for 72h, IL-6 (20 ng/ml) for 24h or pretreated for 16h with milks before starting stimulation for 24h with IL-6. Control cells (Ctr) were treated with the corresponding highest volume of HBSS-10 mM Hepes. Lane 1 = Ctr; lane 2 = IL-6; lane 3 = Hol TMR milk; lane 4 = Hol P milk; lane 5 = Mod P milk; lane 6 = Hol TMR+IL-6; lane 7 = Hol P+IL-6; lane 8 = Mod P+IL-6; M = weight markers (G623, Applied Biological Materials Inc.). Protein expression was calculated, after normalization with α-tubulin or GAPDH as internal control, with IMAGEJ software.

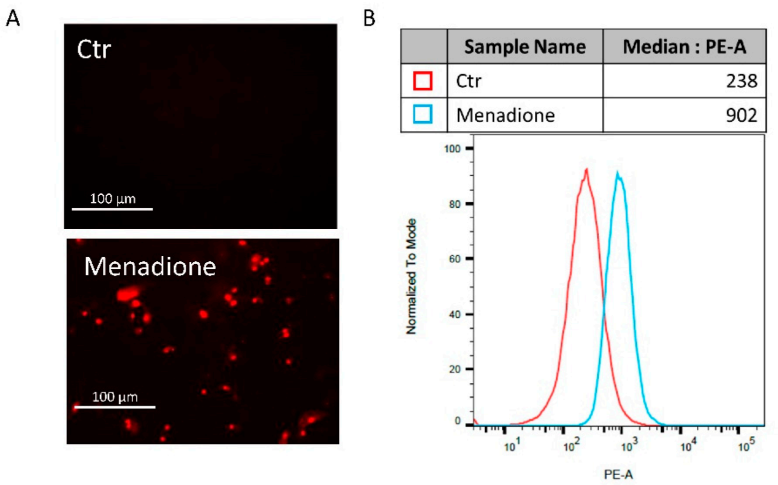

Figure S3. Oxidative stress controls. (A) Representative images and (B) cytometer analysis, expressed as red fluorescence median, of mitochondrial ROS detection in EC treated for 1h with 50 µM menadione, the ROS inducer, or with the corresponding volume of HBSS-10 mM Hepes (Ctr) and stained with MitoSOX Red probe. Scale bar= 100µm.

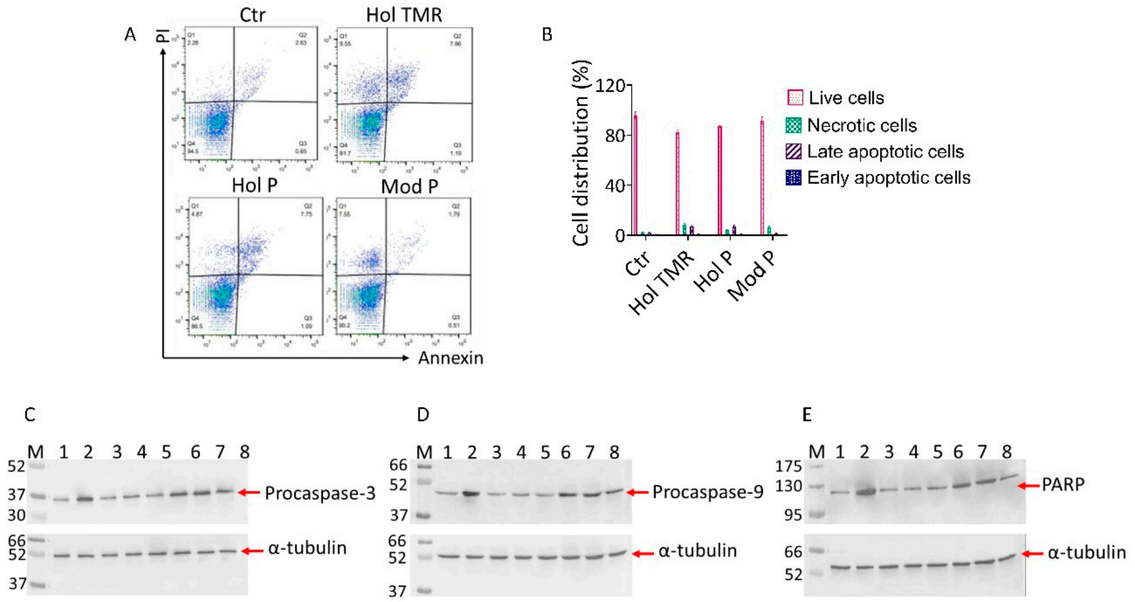

Figure S4. Milk effects on apoptosis. (A) Representative dot plots and (B) analysis of Annexin V-FITC and Propidium Iodide (PI)-stained EC treated with Hol TMR, Hol P, and Mod P milk (20% v/v) for 72h or with the corresponding volume of HBSS-10 mM Hepes (Ctr). Lower left, viable cells; upper left, necrotic cells; lower right, early apoptotic cells; upper right, late apoptotic cells. Representative cropped blots of (C) procaspase-3, (D) procaspase-9 and (E) PARP immunoblotting analysis in EC treated with Hol TMR, Hol P, and Mod P milk (20% v/v) for 72h, IL-6 (20 ng/ml) for 24h or pretreated for 16h with milks before starting stimulation for 24h with IL-6. Control cells (Ctr) were treated with the corresponding highest volume of HBSS-10 mM Hepes. Lane 1 = Ctr; lane 2 = IL-6; lane 3 = Hol TMR milk; lane 4 = Hol P milk; lane 5 = Mod P milk; lane 6 = Hol TMR+IL-6; lane 7 = Hol P+IL-6; lane 8 = Mod P+IL-6; M = weight markers (G623, Applied Biological Materials Inc.). Protein expression was calculated, after normalization with α-tubulin as internal control, with IMAGEJ software.

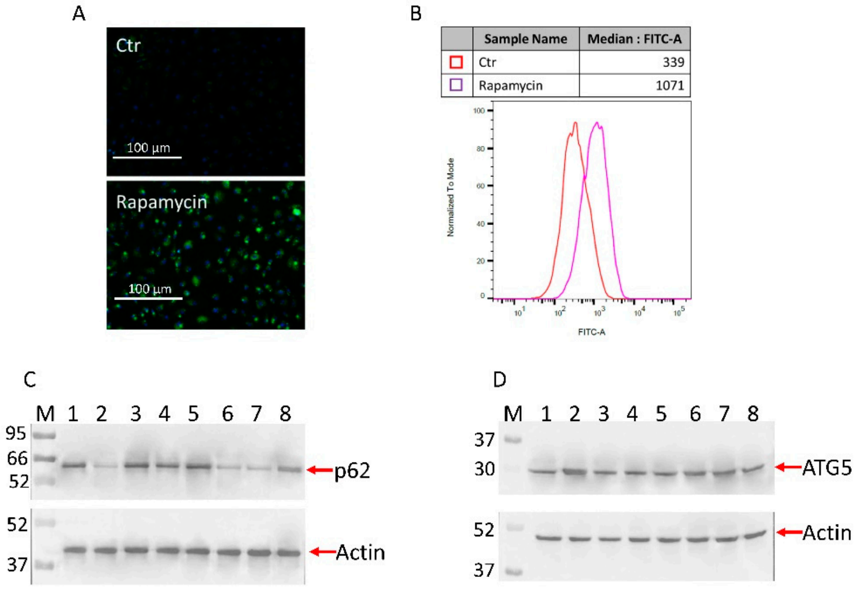

Figure S5. Milk effects on autophagy. (A) Representative images and (B) cytometer analysis, expressed as green fluorescence median, of autophagy detection in EC treated for 16h with 1 µM rapamycin, autophagy inducer, or with the corresponding volume of HBSS-10 mM Hepes (Ctr) and stained with Green detection reagent. Scale bar= 100µm. Representative cropped blots of (C) p62 and (D) ATG5 immunoblotting analysis in EC treated with Hol TMR, Hol P, and Mod P milk (20% v/v) for 72h, IL-6 (20 ng/ml) for 24h or pretreated for 16h with milks before starting stimulation for 24h with IL-6. Control cells (Ctr) were treated with the corresponding highest volume of HBSS-10 mM Hepes. Lane 1 = Ctr; lane 2 = IL-6; lane 3 = Hol TMR milk; lane 4 = Hol P milk; lane 5 = Mod P milk; lane 6 = Hol TMR+IL-6; lane 7 = Hol P+IL-6; lane 8 = Mod P+IL-6; M = weight markers (G623, Applied Biological Materials Inc.). Protein expression was calculated, after normalization with actin as internal control, with IMAGEJ software.
